# Prevalence of human papillomavirus genotypes and precancerous cervical lesions in a screening population in Beijing, China: analysis of results from China’s top 3 hospital, 2009–2019

**DOI:** 10.1186/s12985-020-01383-1

**Published:** 2020-07-13

**Authors:** Yidi Liu, Qing Ang, Huan Wu, Jingjiang Xu, Defu Chen, Hongyou Zhao, Haolin Liu, Xianghuan Guo, Ying Gu, Haixia Qiu

**Affiliations:** 1 Department of Laser Medicine, the First Medical Center, Chinese PLA General Hospital, Beijing, 100853 China; 2 Medical Supplies Center, Chinese PLA General Hospital, Beijing, 100853 China; 3 Medical Big Data Center, Chinese PLA General Hospital, Beijing, 100853 China; 4 School of Physics and Optoelectronic Engineering, Foshan University, Foshan, 528000 China; 5 Institute of Engineering Medicine, Beijing Institute of Technology, Beijing, 100081 China

**Keywords:** Human papilloma virus (HPV), Cervical intraepithelial Neoplasia (CIN), High-grade squamous intraepithelial lesion (HSIL), Low-grade squamous intraepithelial lesion (LSIL)

## Abstract

**Background:**

Cervical cancer is the fourth most common cancer in women. Early detection and diagnosis play an important role in secondary prevention of cervical cancer. This study aims to provide more information to develop an effective strategy for the prevention and control of cervical cancer in northern China.

**Methods:**

A retrospective single-centre descriptive cross-sectional study was conducted in Chinese PLA General Hospital located in Beijing, covering the period from January 2009 to June 2019. The patients who underwent a polymerase chain reaction (PCR)-based HPV genotyping test and cervical pathological diagnosis were included. Furthermore, we limited the interval between the two examination within 180 days for the purpose of making sure their correlation to analyse their relationship. Moreover, the relationship between different cervical lesions and age as well as single/multiple HPV infection was assessed.

**Results:**

A total of 3134 patients were eligible in this study after HPV genotyping test and pathological diagnosis. Most of the patients (95%) were from northern China. Among the patients, 1745(55.68%) had high-grade squamous intraepithelial neoplasia (HSIL), 1354 (43.20%) had low-grade squamous intraepithelial neoplasia (LSIL) and 35 (1.12%) were Normal. The mean age was 42.06 ± 10.82(range, 17–79 years). The women aged 35–49 years accounted for the highest incidence rate. The top five most commonly identified HPV genotypes in each lesion class were as follows: HPV16, 58, 52, 31 and 51 in the class of HSIL; HPV16, 52, 58, 56 and 51 in the class of LSIL; HPV16, 31, 6,11, 52 and 58 in the class of normal. The frequencies of HPV single genotype infection and multiple genotypes infection were 55.26 and 34.18%, respectively. There was no difference in the attributable proportions of multiple genotypes infection amongst HSIL, LSIL and Normal.

**Conclusions:**

In Northern China, HPV16 was the most dominant genotype in the patients with pathological examination. The peak age of the onset of HSIL was between 35 and 49 years of age. Infection with multiple HPV genotypes did not increase the risk of HSIL. Type-specific HPV prevalence and attribution proportion to cervical precancerous lesions should be taken into consideration in the development of vaccines and strategy for screening in this population.

## Introduction

Cervical cancer, a type of malignant genital tract tumour, is the 4th most frequently diagnosed cancer in women worldwide, making it the 4th leading cause of cancer death as well, which greatly threatens women’s lives. According to a report which provided global data and graphical visualization of cancer incidence and mortality using the database of GLOBOCAN 2018 produced by the International Agency for Research on Cancer (IARC), there were 569,847 new cases of cervical cancer causing 311,365 deaths globally in 2018 [1]. In the regions with low/medium Human Development Index (HDI) as well as in China, cervical cancer ranks second for female in incidence and mortality behind breast cancer [1, 2]. Based on the data from World Health Organization [3], in China, cervical cancer was responsible for 106,430 new cases and 47,739 deaths in the year of 2018. According to the existing epidemiological evidence in urban and rural areas of mainland China, if without any intervention, the annual number of new cervical cancer cases is predicted to be dramatically increased, ranging from ∼27,000 to 130,000 in 2010 and reach ∼42,000 to 187,000 in 2050 [4]. Therefore, the prevention and cure of cervical cancer has been one of the major public health problems in China.

Continued infection with the human papillomavirus (HPV) which is known as the most common sexually transmitted virus [5] causes cervical cancer and cervical intraepithelial neoplasia (CIN). Due to this clear relationship, cervical cancer is an avoidable disease that can be prevented, treated and eradicated, compared to many other cancers. According to how much epithelial tissue is affected, CIN can be graded on 1–3 scale, where CIN3 is the most abnormal grade. In this study, CIN1 is equivalent to low-grade squamous intraepithelial neoplasia (LSIL), while ≥CIN2 is called precancerous lesion or high-grade squamous intraepithelial neoplasia (HSIL). CIN1 has the risk of developing into ≥CIN2, and ≥ CIN2 has the risk of developing into cervical cancer. HPV16, 18, 31, 33, 35, 39, 45, 51, 52, 56, 58, 59, and 68 are identified as “high risk HPV” (HR-HPV) due to their relatively high carcinogenic potential leading to the development of cervical cancer among more than 150 HPV strains being found [6].

Currently, large-scale cervical cancer vaccination programs have been launched and have saved many women’s lives [7, 8]. Gardasil® is a commonly used quadrivalent vaccine against HPV6, 11, 16 and 18, while HPV6, 11 are low-risk genotypes that can induce benign genital warts or condylomas [9]. Gardasil 9 was approved by the US Food and Drug Administration (FDA) in 2014 and provided protection against HPV6, 11, 16, 18, 31, 33, 45, 52, and 58 [10]. The preventive effect of HPV vaccine on cervical cancer has been confirmed in multiple studies [11, 12]. However, all HR-HPV strains that can cause cervical cancer are not completely covered by Gardasil 9, and HPV genotype distribution varies between different regions and countries, causing the incidence and mortality of cervical cancer to change geographically as well [13, 14]. Therefore, identifying the distribution of HPV types among different grade cervical lesions will provide baseline information for decisions on HPV vaccination program in China, so that the effectiveness of large-scale vaccination can be assessed and differences in geographical distribution of HPV types can be distinguished. Due to the limitation of HPV examination technology, it is not possible to obtain all types of HPV distribution. Acquiring the distribution of the most carcinogenic HPV types will provide crucial information for developing a new generation of HPV vaccines in line with China’s national conditions. Although many studies have reported the prevalence of HPV genotypes based on the presence of precancerous lesions and invasive cancer [15–17], a reliable and large-population study concerning HPV distribution has been rarely reported in many developing countries or regions previously.

In this retrospective cross-sectional study, patients who had undergone HPV examination and cervical pathological biopsy in the PLA General Hospital from January 2009 to June 2019 were recruited, HPV genotypes were identified, and the distribution of HPV types in different cervical lesions was analysed. The hospital’s comprehensive strength of the PLA General Hospital ranks among the top three hospitals in China in recent years [18]. In addition, the PLA General Hospital is in Beijing which is the capital of China and the most prosperous city in northern China. Thus, most women from northern China prefer to undergo abnormal and opportunistic screening for cervical cancer in this hospital, which provides adequate patient resources to reflect the relationship between HPV genotypes and precancerous cervical lesions. Moreover, the superior clinical and laboratory capabilities in this hospital ensure us to undertake this study. The purpose of this study is to investigate the distribution of HPV types in northern China and their relationship with the degrees of cervical lesions, which provides comprehensive scientific evidence to help develop regional vaccines in the future.

## Methodology

### Study population and samples

**Fig. 1:**
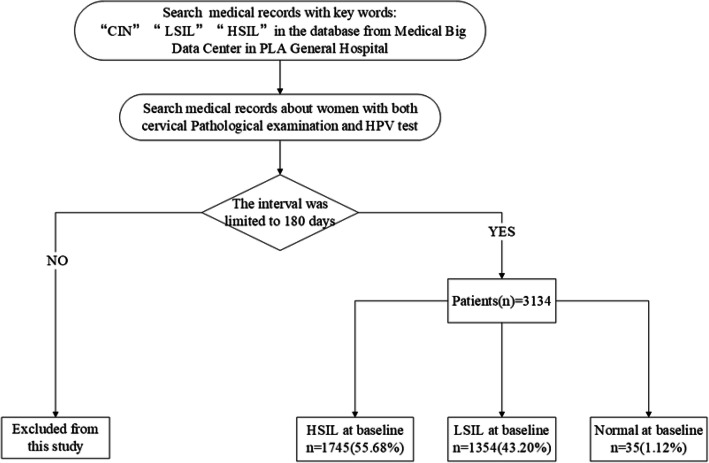
Screening flowchart. CIN, Cervical Intraepithelial Neoplasia; HSIL, high-grade squamous intraepithelial neoplasia; LSIL, low-grade squamous intraepithelial neoplasia; HPV, human papillomavirus

From January 2009 to June 2019, the patients who underwent HPV DNA testing and cervical pathological diagnosis in Chinese PLA general hospital (Beijing, China) were included in this cross-sectional study. Furthermore, we limited the interval between the two examinations within 180 days for the purpose of making sure their correlation to analyze their relationship. (see Fig. 1). Single and multiple HPV infections were assessed according to different cervical cytology. The overall infection rate of specific HPV type and the prevalence of type-specific HPV among different age groups and precancerous lesions were calculated. In this study, according to the severity of the cervical lesion, the study population was divided into 3 groups: HSIL, LSIL, and Normal. Besides, in order to identify the relationship between the age and HPV types as well as cervical lesions, five age groups were set: < 20, 20–34, 35–49, 50–64, 65–79.

The pathological diagnosis of cervical lesions was used as the golden standard. The diagnosis of cervical cytology was classified by the 2001 Bethesda system. Experienced pathologists in the PLA General Hospital reviewed every histology slide and classified each finding as negative, CIN grade 1/2/3. In this study, CIN1 is referred to as low-grade squamous intraepithelial neoplasia (LSIL); CIN 2/3 is regarded as high-grade squamous intraepithelial neoplasia (HSIL).

### Genotype-specific test

DNA extraction and HPV genotyping were carried out using HPV genotyping real-time PCR kit (Shanghai ZJ Bio-Tech Co., Ltd) to detect the following 18 HPV types: HPV6, 11, 16, 18, 21, 31, 33, 35, 39, 45, 51, 52, 56, 58, 59, 66, 67, 68, 82. The lowest detection limit of the kit was 1 × 10^4^ copies/mL. Amplification techniques performed on SLAN®-96P (Shanghai Hongshi Medical Technology Co., Ltd) were used for the quantitative estimation of HPV DNA copies.

### Statistical analysis

A database was established using Excel 2016, and the results were analysed by SPSS 22.0 software (SPSS Inc., Chicago, IL, USA). A Chi-square test was used for the counting analysis, and a t-test was used for variable data. A *p* value of < 0.05 was considered significant.

## Results

### Baseline characteristics

**Fig. 2:**
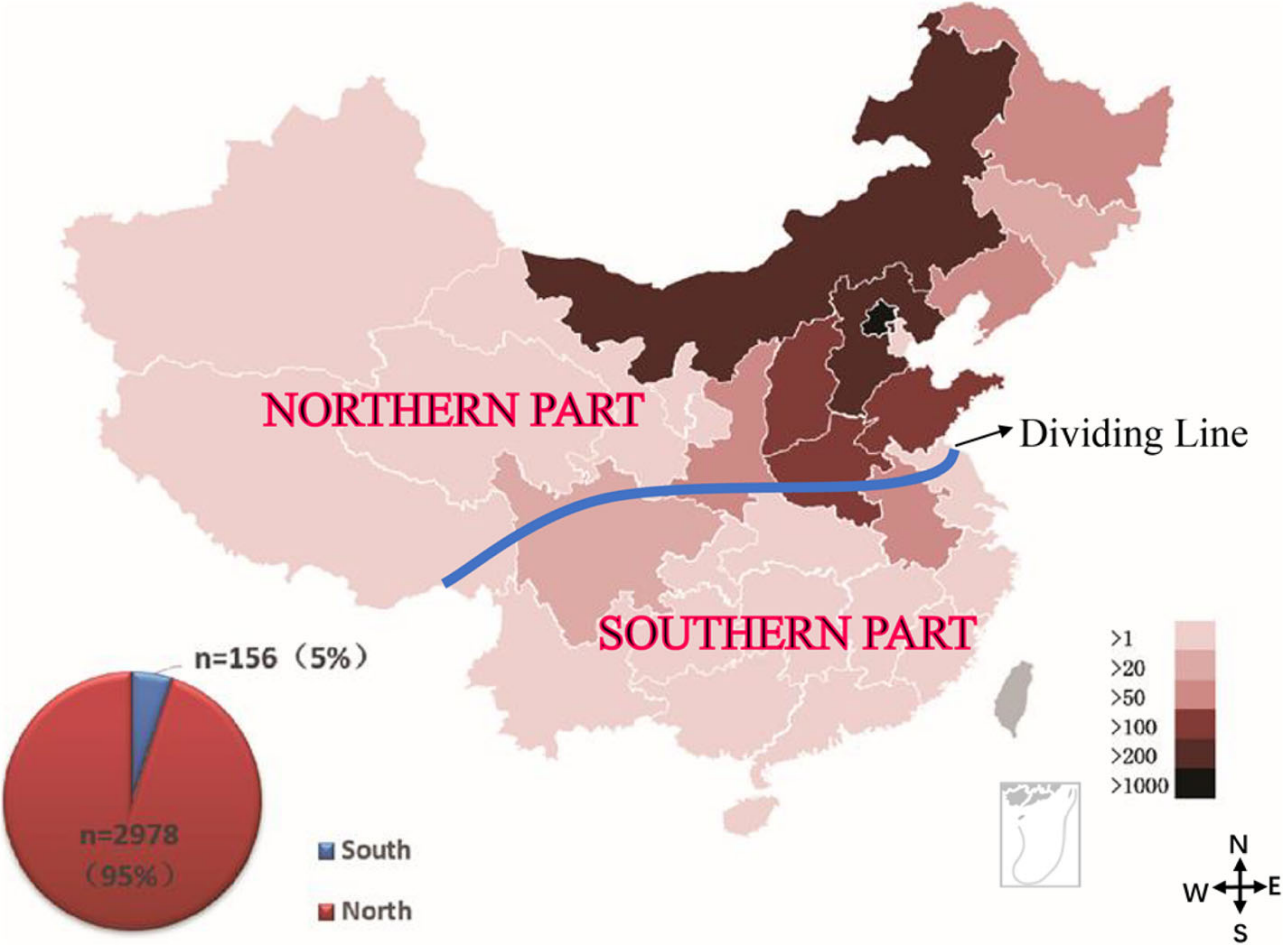
Geographical distribution of the recruited patients (*n* = 3134) in China

In order to analyse the attribution proportion of HPV types to precancerous lesions, there were 3134 eligible patients who underwent histopathological examination after HPV genotype-specific test within 180 days. As shown in Fig. 2, China can be divided into two parts, south and north, along the Qinling Mountains-Huaihe River line. Although the PLA General Hospital attracted the patients all over the country, 95% of the patients enrolled in this study were from northern China due to the location. The mean age of the subjects was 42.06 ± 10.82 years old, where the youngest was 17 years old and the oldest was 79 years old. 3029 (96.65%) were positive for HPV and the 2747 (87.65%) were positive for HR-HPV. The top five HPV genotypes are HPV16, 58, 52, 51 and 56. As for the pathological results, 1745 (55.68%) women had HSIL, 1354 (43.20%) women had LSIL, 35 (1.12%) women had normal cervical cytology. The distributions of age, cervical pathology, HPV genotypes and single/multiple HPV infection among the 3134 patients are presented in Table 1.

**Table 1:** Distribution of age, cervical pathology, HPV prevalence, the relationship between cervical pathology and HPV infection and Single/multiple HPV infection in patients (*n* = 3134)

Characteristics	Mean	Frequency
*Total number*	3134	/
*Age (yr)*		
< 20	3	0.10%
20–34	880	28.08%
35–49	1480	47.22%
50–64	681	21.73%
65–80	90	2.87%
*Cervical cytology*		
HSIL (CIN 2+)	1745	55.68%
LSIL (CIN1)	1354	43.20%
Normal	35	1.12%
*HPV*		
HPV6,11	104	2.32%
HPV16	1223	27.34%
HPV18	252	5.63%
HPV21	52	1.16%
HPV31	196	4.38%
HPV33	169	3.78%
HPV35	111	2.48%
HPV39	198	4.43%
HPV45	64	1.43%
HPV51	283	6.33%
HPV52	532	11.89%
HPV56	273	6.10%
HPV58	560	12.52%
HPV59	141	3.15%
HPV66	114	2.55%
HPV67	31	0.69%
HPV68	122	2.73%
HPV82	49	1.10%
*Cervical cytology/HPV*		
HSIL (HPV+)	1640	52.33%
HISL (HPV-)	105	3.35%
LSIL (HPV+)	1195	38.13%
LSIL (HPV-)	159	5.07%
Normal (HPV+)	20	0.64%
Normal (HPV-)	15	0.48%
*HPV Single infection*	1784	56.92%
*HPV Multiple infection*	1071	34.17%

### Age distribution of patients with different HPV-type infections

**Fig. 3:**
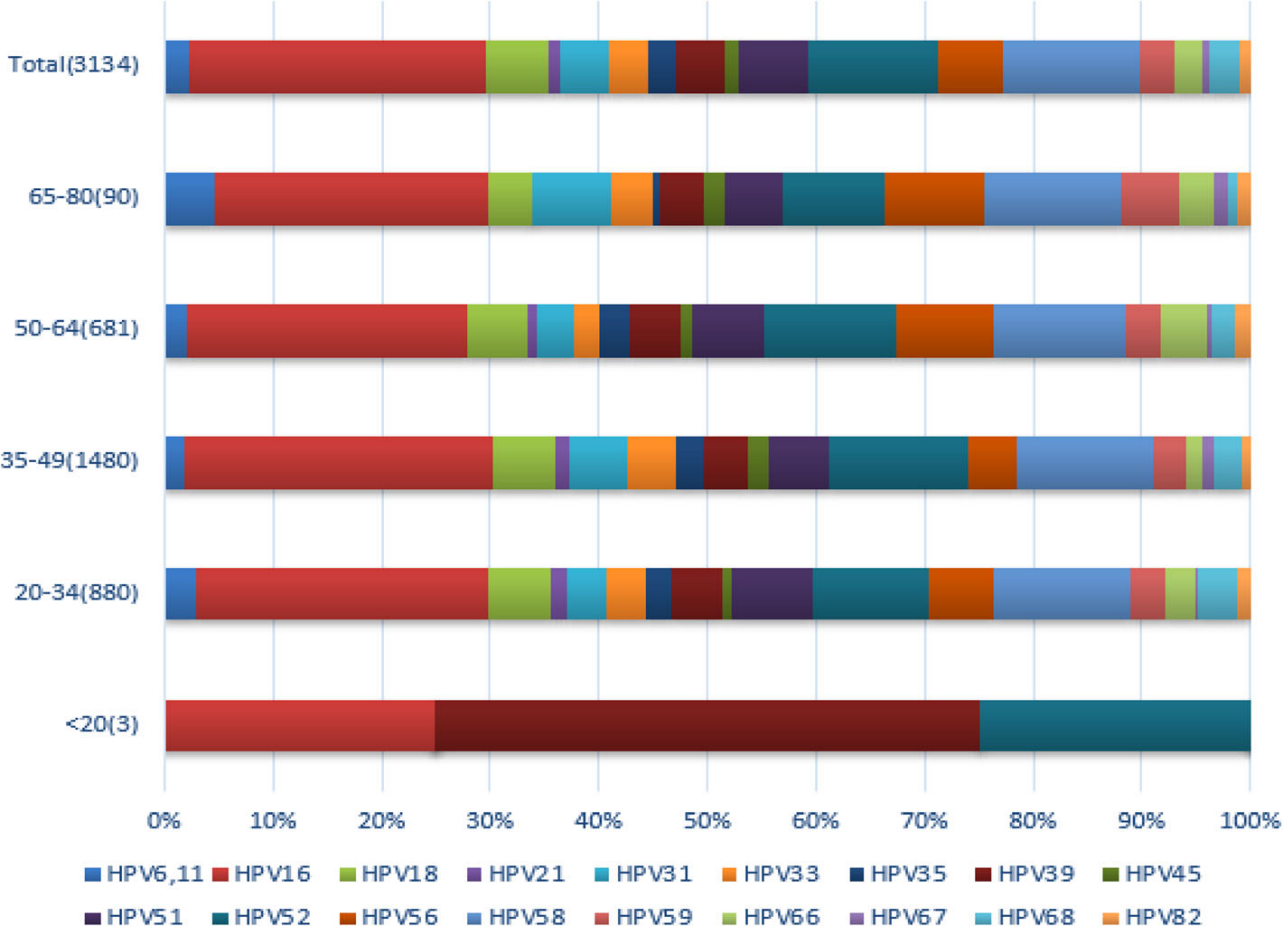
Age-stratified HPV distribution in patients (n = 3134, including multiple-genotype infection, HPV6, 11, 16, 18, 21, 31, 33, 35, 39, 45, 51, 52, 56, 58, 59, 66, 67, 68 and 82 (%))

Age-stratified HPV distribution of the patients in the study is shown in Table 2 and Fig. 3. In the group of < 20 age, there were only 3 eligible patients and one of them was infected by two HPV types, leading to 25, 50 and 25% infection rate for HPV16, 39 and 58, respectively. For the patients at the age of 20–34, the top five HPV genotypes were HPV16 (26.91%), 58 (12.54%), 52 (10.70%), 51 (7.33%) and 56 (6.09%); at the age of 35–49, the top five genotypes were HPV16 (28.51%), 52 (12.76%), 58 (12.71%), 18 (5.77%) and 51 (5.57%); at the age of 50–64, the top five genotypes were HPV16 (25.92%), 58 (12.14%), 52 (12.14%), 56 (8.88%) and 51 (6.63%); at the age of 65–80, the top five genotypes were HPV16 (25.17%), 58 (12.58%), 52(9.27%), 56(9.27%), 31(7.28%). In the patients at the age of 20–34 and 50–64, the overall prevalence of the HPV genotypes was quite similar where the top five HPV genotypes were the same but with slightly different order. However, with the regard to the 35–49 age group, HPV 18 which was not among the top 5 HPV infection types while accounted for 5.77%; at the age group 65–80, HPV 31 which also was not among the top 5 HPV infection types while occupied for 7.28%. Moreover, the prevalence of HPV16 was the highest among all the age groups except for the group of < 20 in which the sample size was too small.

**Table 2:** Age-stratified HPV distribution (15-year interval) in patients (*n* = 3134)

HPV Type/Age	< 20		20–34		35–49		50–64		65–80		Total	
	n	%	n	%	n	%	n	%	n	%	n	%
HPV6,11	0	0.00%	41	3.01%	36	1.82%	20	2.04%	7	4.64%	104	2.32%
HPV16	1	25.00%	367	26.91%	563	28.51%	254	25.92%	38	25.17%	1223	27.34%
HPV18	0	0.00%	78	5.72%	114	5.77%	54	5.51%	6	3.97%	252	5.63%
HPV21	0	0.00%	21	1.54%	23	1.16%	8	0.82%	0	0.00%	52	1.16%
HPV31	0	0.00%	48	3.52%	104	5.27%	33	3.37%	11	7.28%	196	4.38%
HPV33	0	0.00%	50	3.67%	89	4.51%	24	2.45%	6	3.97%	169	3.78%
HPV35	0	0.00%	31	2.27%	52	2.63%	27	2.76%	1	0.66%	111	2.48%
HPV39	2	50.00%	66	4.84%	79	4.00%	45	4.59%	6	3.97%	198	4.43%
HPV45	0	0.00%	11	0.81%	38	1.92%	12	1.22%	3	1.99%	64	1.43%
HPV51	0	0.00%	100	7.33%	110	5.57%	65	6.63%	8	5.30%	283	6.33%
HPV52	1	25.00%	146	10.70%	252	12.76%	119	12.14%	14	9.27%	532	11.89%
HPV56	0	0.00%	83	6.09%	89	4.51%	87	8.88%	14	9.27%	273	6.10%
HPV58	0	0.00%	171	12.54%	251	12.71%	119	12.14%	19	12.58%	560	12.52%
HPV59	0	0.00%	45	3.30%	56	2.84%	32	3.27%	8	5.30%	141	3.15%
HPV66	0	0.00%	38	2.79%	30	1.52%	41	4.18%	5	3.31%	114	2.55%
HPV67	0	0.00%	2	0.15%	23	1.16%	4	0.41%	2	1.32%	31	0.69%
HPV68	0	0.00%	50	3.67%	49	2.48%	22	2.24%	1	0.66%	122	2.73%
HPV82	0	0.00%	16	1.17%	17	0.86%	14	1.43%	2	1.32%	49	1.10%
Total	4	100.0%	1364	100.0%	1975	100%	980	100.0%	151	100.0%	4474	100.0%

### Age distribution of patients in different grades of cervical lesions

**Fig. 4:**
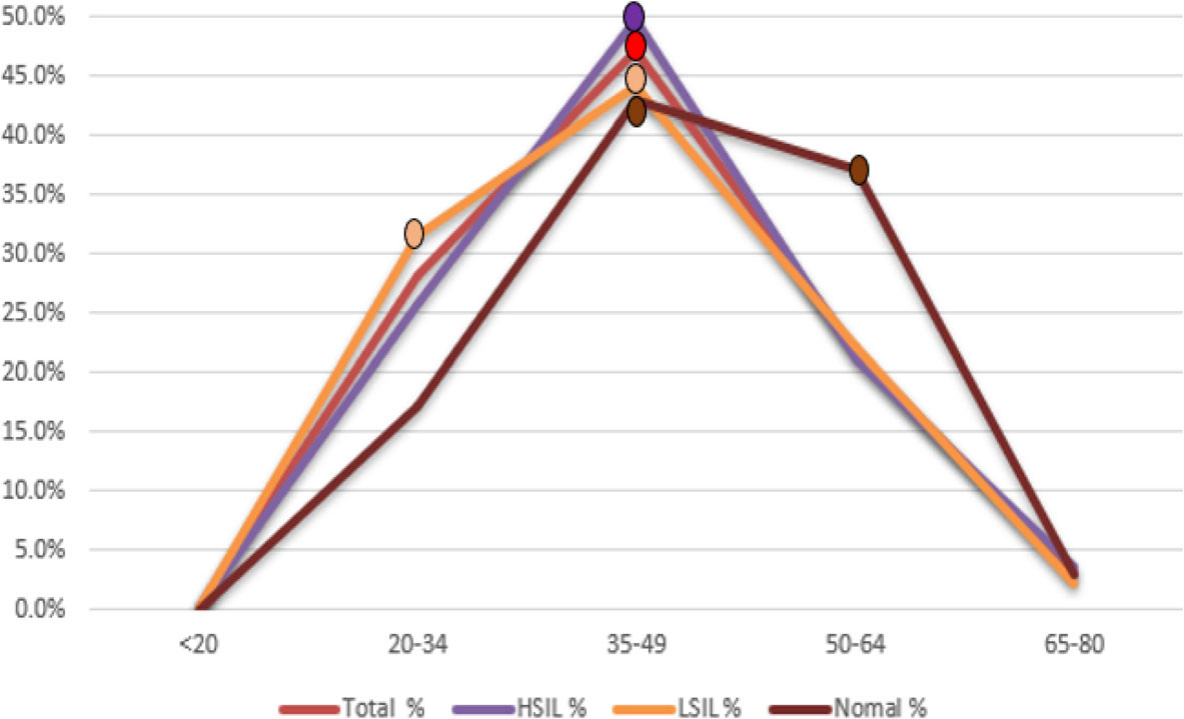
Age distribution of different grade of cervical lesions

In this study, the prevalence of HSIL reached a peak in patients between 35 and 49 years of age, which was later than the peak for LSIL lesions. Age-stratified distribution of different cervical lesions in the study were presented by Table 3. To clearly show the results, a line chart of the age distribution is shown in Fig. 4. As we can see from Fig.4, among the group HSIL, LSIL, Normal, the peak age was at 35–49 years old and the rate was 49.70, 44.20, 42.90% respectively. However, as for the group LSIL, besides group 35–49 years old, the group 20–34 years old also has relatively high rate of 31.50%; in terms of group Normal, besides group 35–49 years old, the group 50–64 years old has second highest rate of 37.10%. The prevalence of LSIL demonstrated two peaks at 20–34 and 35–49, where there was no statistical significance between 20 and 34 and 35–49. The peak age of the onset of LSIL was at 20–34 years of age, which was around 7 years earlier than that of HSIL which had a peak at 35–49 years of age. The patients with normal cervix lesions also showed two peaks at 35–49 and 50–64 (there was no statistical significance between 35 and 49 and 50–64).

**Table 3:** Age distribution of different grade of cervical lesions (*n* = 3134)

Age (years)	HSIL		LSIL		Normal		Total	
	n	%	n	%	n	%	n	%
< 20	1	0.10%	2	0.10%	0	0.00%	3	0.10%
20–34	448	25.70%	426	31.50%	6	17.10%	880	28.10%
35–49	867	49.70%	598	44.20%	15	42.90%	1480	47.20%
50–64	369	21.10%	299	22.10%	13	37.10%	681	21.70%
65–80	60	3.40%	29	2.10%	1	2.90%	90	2.90%
Total	1745	100%	1354	100%	35	100%	3134	100%

The proportion of year between 35 and 49 in HSIL was significantly different from that of other age group, *P* < 0.01 (< 20, χ2 = 66.67; 20–34, χ2 = 12.22; 50–64, χ2 = 18.36; 65–80, χ2 = 56.71)

The proportion of year between 35 and 49 in LSIL was significantly different from that of other age group, *P* < 0.01 (< 20, χ2 = 56.41; 50–64, χ2 = 10.95; 65–80, χ2 = 49.80) except for 20–34, χ2 = 3.242, *P* = 0.072 > 0.05

The proportion of year between 35 and 49 in Normal is significantly different from that of other age group, *P* < 0.01 (20–34, χ2 = 16.10; 50–64, χ2 = 0. 75, *P* = 0.386; 65–80, χ2 = 45.17) except for 50–64, χ2 = 0.75, *P* = 0.386 > 0.05

### Frequency of infection with a single or multiple HPV genotypes in cervical lesions

**Fig. 5:**
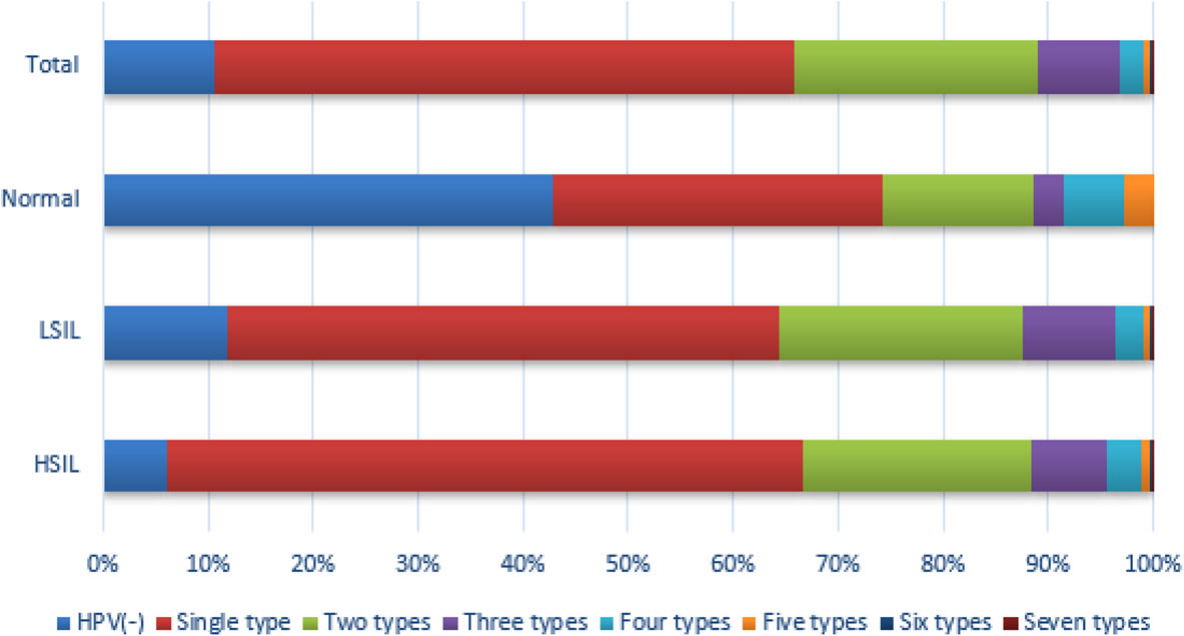
Stratified- multiple HPV genotypes in different cervical pathological results HPV (−), (HPV) Single type, Two types, Three types, Four types, Five types, Six types (%)

As shown in Table 4, in all of HPV infected patients, infection with one, two, three, four, five, six, and seven genotypes of HPV was detected in 1732 cases, 726 cases, 248 cases, 65 cases, 22 cases, 8 cases and 2 cases, respectively. The frequency of a single HPV genotype infection was 55.26%, while that of multiple genotypes was 34.18%. Infection with two different genotypes was the most common multiple HPV infections, where the maximum multiple infections were seven genotypes. Among HSIL patients, infection with single, 2, 3, 4, 5, 6, 7 genotypes accounted for 60.7, 21.6, 7.3, 3.3, 0.8, 0.2 and 0.1%, respectively. As the number of HPV genotypes increased, the attributable proportion to HSIL decreased (shown in Table 3). There were no statistical differences in the frequencies of multiple HPV genotypes amongst different cervical lesions, suggesting that increased numbers of HPV genotypes did not increase the risk for HSIL. As illustrated by Fig. 5, the distribution of different genotypes to HSIL, LSIL, Normal has been presented.

**Table 4:** Frequency of multiple HPV types in women with different precancerous grades

HPV genotypes	HSIL		LSIL		Normal		Total	
	n	%	n	%	n	%	n	%
HPV (−)	105	6.02%	159	11.74%	15	42.86%	331	10.56%
Single type	1059	60.69%	714	52.73%	11	31.43%	1732	55.26%
Two types	377	21.60%	313	23.12%	5	14.29%	726	23.17%
Three types	127	7.28%	120	8.86%	1	2.86%	248	7.91%
Four types	58	3.32%	36	2.66%	2	5.71%	65	2.07%
Five types	14	0.80%	7	0.52%	1	2.86%	22	0.70%
Six types	4	0.23%	4	0.30%			8	0.26%
Seven types	1	0.06%	1	0.07%			2	0.06%
Total	1745	55.68%	1354	43.20%	35	1.12%	3134	100.00%

The proportion of HPV (−) in Normal is significantly different from that of other groups, *P* < 0.05 (HSIL, χ2 = 37.05; LSIL, χ2 = 24.10)

The proportion of Single type in HSIL is not significantly different from that of other groups, *p* > 0.05 (HSIL, χ2 = 1.31) except for Normal, (χ2 = 18.12, *P* < 0.05)

The proportion of Two types in HSIL is not significantly different from that of other groups *P* > 0.05 (LSIL, χ2 = 0.03; Normal, χ2 = 2.17)

The proportion of Three types in HSIL is not significantly different from that of other groups *P* > 0.05 (LSIL, χ2 = 0.27; Normal, χ2 = 1.68)

The proportion of Four types in HSIL is not significantly different from that of other groups *P* > 0.05 (LSIL, χ2 = 0.00; Normal, χ2 = 1.05)

The proportion of Five types in HSIL is not significantly different from that of other groups *P* > 0.05 (LSIL, χ2 = 0.00; Normal, χ2 = 1.02)

The proportion of Six types in HSIL is not significantly different from that of other groups *P* > 0.05 (LSIL, χ2 = 0.00)

The proportion of Seven types in HSIL is not significantly different from that of other groups *P* > 0.05 (LSIL, χ2 = 0.00)

### Distribution of HPV genotypes in women with different cervical lesions

Among the 3134 patients underwent histopathological examination after HPV test within 180 days, there were 3, 099 precancerous cases and 35 normal cases. 3029 (96.64%) cases were positive for HPV and 2747 (87.65%) cases were positive for HR-HPV. According to Table 5, in the group of HSIL, HPV16 (56.46%) was the most frequent genotype, followed by HPV58 (18.41%), 52 (16.22%), 31 (8.23%) and 51 (7.68%). It is crucial to note that HPV16 was attributed more to HSIL than HPV52 and 58. Moreover, there was no significant difference between the distribution of HPV52 and 58. HPV16 (24.18%), 52 (22.01%), 58 (21.34%),56 (14.23%) and 51 (13.05%) were the most frequently detected types in LSIL, and there was no statistical difference for these former four types in the attribution to LSIL. The percentage of cases of with HPV 16 detected was 56.46% and was markedly higher in the HSIL group. As for the patients with normal cervix, HPV16 was also the dominated HPV genotype which accounted for 40.0%. HPV31 (25%), 6,11 (20%), 52 (15%), 58 (15%) and 66 (15%) were also the common types detected in normal cytology cases.

**Table 5:** Distribution of HPV infection with a single genotype and multiple genotypes in women (n = 3134)

Types	HSIL		LSIL		Normal		Total	
	n	%	n	%	n	%	n	%
Total	1745		1354		35		3134	
Total HPV (+)	1640	93.98%	1195	88.26%	20	57.14%	2803	89.44%
HPV6,11	36	2.20%	64	5.36%	4	20.00%	104	3.71%
HPV16	926	56.46%	289	24.18%	8	40.00%	1223	43.63%
HPV18	120	7.32%	132	11.05%			252	8.99%
HPV21	38	2.32%	14	1.17%			52	1.86%
HPV31	135	8.23%	56	4.69%	5	25.00%	196	6.99%
HPV33	123	7.50%	45	3.77%	1	5.00%	169	6.03%
HPV35	53	3.23%	57	4.77%	1	5.00%	111	3.96%
HPV39	70	4.27%	128	10.71%			198	7.06%
HPV45	36	2.20%	26	2.18%	2	10.00%	64	2.28%
HPV51	126	7.68%	156	13.05%	1	5.00%	283	10.10%
HPV52	266	16.22%	263	22.01%	3	15.00%	532	18.98%
HPV56	102	6.22%	170	14.23%	1	5.00%	273	9.74%
HPV58	302	18.41%	255	21.34%	3	15.00%	560	19.98%
HPV59	71	4.33%	68	5.69%	2	10.00%	141	5.03%
HPV66	40	2.44%	71	5.94%	3	15.00%	114	4.07%
HPV67	22	1.34%	7	0.59%	2	10.00%		
HPV68	34	2.07%	87	7.28%	1	5.00%	122	4.35%
HPV82	27	1.65%	22	1.84%			49	1.75%

The proportion of HPV16 type in HSIL was significantly different from that of other high-risk type HPV, *P* < 0.05 (HPV58, χ2 = 30.97; HPV52, χ2 = 34.72; HPV31, χ2 = 52.94; HPV51, χ2 = 52.94)

The proportion of HPV58 type in HSIL was significantly different from that of other high-risk type HPV, *p* < 0.05 (HPV16, χ2 = 30.97; HPV31, χ2 = 4.42; HPV51, χ2 = 4.42) except for HPV52, χ2 = 0.14, *p* = 0.71 > 0.05

The proportion of HPV52 type in HSIL was significantly different from that of HPV16, χ2 = 34.72, *p* < 0.05 except for HPV58, χ2 = 0.14, *p* = 0.71 > 0.05; HPV31, χ2 = 3.03, *p* = 0.08 > 0.05; HPV51, χ2 = 3.03, *p* = 0.08 > 0.05

The proportion of HPV16 type in LSIL was significantly different from that of HPV51, χ2 = 4.01, *p* = 0.045 < 0.05 except for HPV52, χ2 = 0.11, *p* = 0.72 > 0.05; HPV58, χ2 = 0.26, *p* = 0.611 > 0.05; HPV56, χ2 = 3.25, *p* = 0.071 > 0.05

The proportion of HPV52 type in LSIL was not significantly different from that of following HPV type, *P* > 0.05 (HPV16, χ2 = 0.11, *p* = 0. 72; HPV58, χ2 = 0.03, *p* = 0.86; HPV56, χ2 = 2.17, *p* = 0.14; HPV51, χ2 = 2.91, *p* = 0.088

The proportion of HPV58 type in LSIL was not significantly different from that of following HPV type, *P* > 0.05 (HPV58, χ2 = 0.26, *p* = 0.61; HPV52, χ2 = 0.03, *p* = 0.86; HPV56, χ2 = 1.70, *p* = 0.19; HPV51, χ2 = 2.27, *p* = 0.13

The proportion of HPV16 type in normal cervical lesion was significantly different from that of following HPV type, *P* < 0.05 (HPV31, χ2 = 5.13, *p* = 0.024; HPV6,11,52,58,66, Fisher test, *p* < 0.0001;)

The proportion of HPV31 type in normal cervical lesion was significantly different from HPV16, χ2 = 5.13, *p* = 0.024 < 0.05; except for HPV6,11,52,58,66 (Fisher test, *p* = 0.111 > 0.05

## Discussion

In this study, despite that our study population had geographical limitations as a single institutional survey, most of them came from northern China due to the location of PLA General Hospital (i.e., Beijing), thus providing representative samples of northern Chinese women in general. In addition, this was a long-term study, lasting for 10 years from January 2009 to June 2019, which provided the most current data for a large population in Northern China.

Among the population which both received HPV and pathological examination, the peak age of onset of precancerous lesion was between 35 and 49 years, while the previous study [19] published in 2017 had a peak age between 30 and 39 years. As for the age distribution of the population in the two studies, there was no statistically difference, where the mean age in our study was 42.06 ± 10.82 years old and the average age in that study of [20] was 40.93 ± 11.87 years old. The peak age of onset of HSIL in our study was around 7 years later than that in that study [20], probably due to the different parts of China and an increasing attention on preventing cervical cancer in recent years. Moreover, the single/multiple HPV infection is also associated with the levels of cervical precancerous lesions. Single HPV infection had the highest prevalence among HSIL/LSIL and there were no differences in the frequencies of multiple types amongst different cervical lesions, suggesting that increased numbers of HPV types did not increase the risk for HSIL, which was consistent with the studies of [20, 21].

HPV16 was the most common type in patients of HSIL (CIN2+) which was the most severe classification in this study. In this study, HPV16 had the highest prevalence amongst different levels of cervical precancerous lesions: HSIL (56.46%, 926/1640); LSIL (24.18%, 289/1195); Normal (40%, 8/20). In other studies, HPV16 was also the most common type in patients with precancerous neoplasia lesions both in Pishan county, Shenyang city, Shenzhen city and other provinces in China [22–24] and worldwide [15, 25, 26]. However, some other studies found that HPV52 was the most common genotype in rural North China [27] and Jiangsu, Guangdong and other provinces in China [28, 29]. All these studies indicated that HPV genotype distributions varied between different regions.

In this study, although HPV52 or 58 was not the most detected type compared to HPV16, the sum of the distribution of HPV52 and 58 was approximately as much as that of HPV16, which should deserve special attention. According to the statistical data, HPV16, 58, 52 combined were attributed to 91.1% of all HSIL (CIN2+) lesions; HPV58 and HPV52 were attributed to 34.6% of HSIL (CIN2+) cases. Nevertheless, in accordance with updated cervical cancer screening guidelines [30], only women who were HPV 16/18 positive were recommended to undergo colposcopy directly, and women who were not HPV16/18 positive were referred to cytology first, and then colposcopy if the cytology was abnormal. However, according to the above discussion, special attention should be paid on HPV58 and 52 in screening procedures in Northern China. Combining specific types of HPV with attributable proportions of HSIL can provide more accurate and effective information for cervical cancer screening programs in specific regions. Currently, there are bivalent Cervarix® and both quadrivalent and 9-valent Gardasil® approved by National Medical Products Administration of China. However, the HPV vaccine has not been added into the National Immunization Program. Recently, the National Infectious Disease Diagnostic Reagent and Vaccine Engineering Technology Research Centre of Xiamen University has developed the first domestically produced bivalent HPV vaccine against HPV16 and 18, which is expected to reduce the patients’ economic burden with a relatively cheap price. This study is supposed to provide important database which will benefit the further development of national multivalent HPV vaccine.

It is critical to note that the overall distribution and attribution proportion of each HPV genotypes elsewhere in the world are not quite similar as that of the Northern Chinese population in this study. HPV16, 18 and 45 were the most prevalent types of HPV associated with cervical lesions from a worldwide-pooled study [31]. In consistent with the above study, we also showed that HPV16 was attributed to the highest rates in HSIL (CIN2+). However, there existed some obvious differences between the worldwide-pooled study [31] and our study: 1) HPV18 had higher infection rate than HPV58 and 52 worldwide which was not true in our study; 2) HPV52 and HPV58 were more frequently detected instead of HPV45 in our study. 3) HPV51 and HPV56 as 9.03, 8.71% of the examined women were also positive for these variants, which is more than the 8.04% for HPV18. There were three possible reasons: first, here we did not investigate the prevalence of HPV types in invasive cervical cancer, HPV18 was more frequently present in adenocarcinoma rather than in precancerous lesions [32, 33]; second, HPV58 was more frequently detected in Asian cervical cancer cases than in Europe or Africa; three, the distribution and attribution of the HPV types vary geographically, not only in different parts of China but also in different regions around the world.

## Conclusion

In Northern China, this study suggested that the peak age of the onset of HSIL/LSIL was between 35 and 49 years of age. Infection with multiple high-risk HPV types did not increase the risk for high-grade squamous intraepithelial lesion. HPV16, HPV58, and HPV52 were the most dominant high-risk genotypes which attributed to 91.1% of all HSIL (CIN2+) lesions. Besides HPV58 and 52, HPV51 and HPV56 also should be taken into consideration when developing vaccination program in Northern China. In terms of further developing national multivalent HPV vaccine program, the results of this study will contribute to provide critical epidemiological evidence and baseline data.

## Data Availability

All data and materials described in the manuscript are available.

## Abbreviations

HPV: Human papillomavirus
HR-HPV: High risk Human papillomavirus
CIN: Cervical Intraepithelial Neoplasia
HSIL: High-grade squamous intraepithelial lesion
LSIL: Low-grade squamous intraepithelial lesion
